# Functional implications of *Drosophila* insulin-like peptides in metabolism, aging, and dietary restriction

**DOI:** 10.3389/fphys.2013.00288

**Published:** 2013-10-16

**Authors:** Kavitha Kannan, Yih-Woei C. Fridell

**Affiliations:** ^1^Department of Molecular and Cell Biology, University of Connecticut-Storrs, Storrs, CT, USA; ^2^Department of Allied Health Sciences, University of Connecticut-Storrs, Storrs, CT, USA

**Keywords:** *Drosophila* insulin-like peptides, insulin-like peptide producing cells, lifespan, metabolism, dietary restriction, dFOXO, dSir2

## Abstract

The neuroendocrine architecture and insulin/insulin-like signaling (IIS) events in *Drosophila* are remarkably conserved. As IIS pathway governs growth and development, metabolism, reproduction, stress response, and longevity; temporal, spatial, and nutrient regulation of *dilp*s encoding *Drosophila* insulin-like peptides (DILPs) provides potential mechanisms in modulating IIS. Of eight DILPs (DILP1–8) identified, recent studies have furthered our understanding of physiological roles of DILP2, DILP3, DILP5, and DILP6 in metabolism, aging, and responses to dietary restriction (DR), which will be the focus of this review. While the DILP producing IPCs of the brain secrete DILP2, 3, and 5, fat body produces DILP6. Identification of factors that influence *dilp* expression and DILP secretion has provided insight into the intricate regulatory mechanisms underlying transcriptional regulation of those genes and the activity of each peptide. Studies involving loss-of-function *dilp* mutations have defined the roles of DILP2 and DILP6 in carbohydrate and lipid metabolism, respectively. While DILP3 has been implicated to modulate lipid metabolism, a metabolic role for DILP5 is yet to be determined. Loss of *dilp2* or adult fat body specific expression of *dilp6* has been shown to extend lifespan, establishing their roles in longevity regulation. The exact role of DILP3 in aging awaits further clarification. While DILP5 has been shown associated with DR-mediated lifespan extension, contradictory evidence that precludes a direct involvement of DILP5 in DR exists. This review highlights recent findings on the importance of conserved DILPs in metabolic homeostasis, DR, and aging, providing strong evidence for the use of DILPs in modeling metabolic disorders such as diabetes and hyperinsulinemia in the fly that could further our understanding of the underlying processes and identify therapeutic strategies to treat them.

## Introduction

Evolutionarily conserved insulin/insulin-like growth factor signaling (IIS) pathway governs growth and development, metabolism, reproduction, stress response, and longevity. In *Drosophila*, eight insulin-like peptides (DILPs) and one insulin receptor (DInR) are found. DILPs 1–8 have been identified mostly through their sequence homology to the mammalian insulin and the typical B-C-A domain structure as observed in mammalian insulin (Gronke and Partridge, 2010). Early biochemical studies confirmed tyrosine phosphorylation of the DInR stimulated by DILP2- or DILP5-containing conditioned media (Rulifson et al., 2002). Functional conservation of DILP5 was recently revealed where DILP5 binds to and activates the human insulin receptor in lowering circulating glucose levels (Sajid et al., 2011). Furthermore, altered expression of genes encoding DILP2, 3, 5, and 6 results in modulated IIS and profound metabolic and longevity consequences (Broughton et al., 2005, 2008; Gronke et al., 2010; Bai et al., 2012). In this review, we will discuss recent progress on our understanding of the diverse biological roles of DILPs in metabolic control, dietary restriction (DR), and lifespan, with a focus on DILPs 2, 3, 5, and 6 given available emerging research findings. Consistent with the broad and diverse physiological consequences of IIS, specific temporal, and spatial expression patterns of individual *dilps* suggest potentially specialized interactions between each DILP and the DInR. Furthermore, we will discuss the regulation, functional diversity, and redundancy of the DILPs as circulating peptides and the physiology of the tissues producing them. Recent discoveries of the involvement of the nutrient sensing fat body in controlling DILP secretion from insulin-like peptide producing cells (IPCs) in the brain has provided a physiological link between those two major tissues governing nutrient sensing, metabolism, and aging (Geminard et al., 2009; Bai et al., 2012; Rajan and Perrimon, 2012). Finally, we will discuss how DILPs are modulated under DR and how such regulation affects the lifespan of the organism.

## Nutrient, temporal, and spatial regulation of *dilp* expression and dilp secretion

More than a decade ago, the search for the extracellular ligands for the DInR led to the identification of seven *Drosophila insulin-like peptide* genes *(dilp*1–7) with diverse temporal and spatial specific expression patterns (Brogiolo et al., 2001; Ikeya et al., 2002; Rulifson et al., 2002). The newest member *dilp8*, has recently been added to the family (Colombani et al., 2012; Garelli et al., 2012). During development, while *dilp2*, *dilp4*, and *dilp7* transcripts are detected in midgut and mesoderm during late-stage embryogenesis, transcripts of *dilp3*, *dilp5*, or *dilp6* are not detectable until larval stages (Brogiolo et al., 2001). In larvae, low levels of *dilp2* expression are detected in the imaginal discs whereas a high signal is measured in seven IPCs of each brain hemisphere and in salivary glands (Brogiolo et al., 2001). Similarly, *dilp5* transcripts, turned on in the second instar and *dilp3* transcripts measured in the mid to late third instar are both detected in the brain IPCs (Ikeya et al., 2002). Recent reports have revealed that *dilp5* is a transcriptional target of a synergistic interaction between Eyeless and Dachshund (Clements et al., 2008; Okamoto et al., 2012). *dilp6* is highly expressed in the larval fat body whereas low levels of its expression are detected in gut and brain (Slaidina et al., 2009). Recent reports demonstrated *dilp8* expression detected in larval imaginal discs (Colombani et al., 2012; Garelli et al., 2012). In the adult stage, expression of *dilps2*, *3,* and *5* but not *dilp1* is detected in IPCs (Broughton et al., 2005). In addition to its expression in IPCs, *dilp5* transcripts are also detected in follicle cells of stage 10 oocytes (Ikeya et al., 2002) and *dilp3* mRNA found in muscle cells of the midgut (Veenstra et al., 2008). Adult expression of *dilp4* is not known (Gronke and Partridge, 2010). Transcripts of *dilp6* are measured most abundantly in the adult fat body and at lower levels in head carcass and brain (Bai et al., 2012). Finally, transcripts of *dilp*7 are detected in specific neurons of the ventral cord (dMP2) and several neurons in the brain (Miguel-Aliaga et al., 2008).

The critical roles of DILPs in animal development and energy homeostasis are evidenced by the fact that their expression is not only regulated temporally and spatially during development but also by nutrient status. As DInR activity is reduced following starvation, it was posited that this could be due to lack of DILPs under low nutrient availability (Britton et al., 2002). Indeed, upon starvation, expression levels of *dilp3* and *dilp5*, but not *dilp2* are reduced (Ikeya et al., 2002). A recent study demonstrated a role of dSir2, the *Drosophila* homolog of mammalian histone deacetylase SIRT1 in regulating the expression of *dilp2* and *dilp5* where systemic knockdown of *dSir2* up-regulates those two *dilps* (Banerjee et al., 2012). In addition, fat body-specific knockdown of *dSir2* is sufficient to up-regulate *dilp2* and *dilp5* expression with changes in *dilp3* transcript levels in those flies not reported (Banerjee et al., 2012, 2013). Finally, dSir2-mediated regulation of these two *dilps* is shown to act independently of dFOXO, a forkhead box-O transcription factor. Transcript levels of *dilp2* and *dilp5* were up-regulated in flies expressing both *dSir2 RNAi* and *dFOXO-TM* (constitutively active dFOXO) constructs in their fat body similar to the levels observed in *dSir2 RNAi* flies (Banerjee et al., 2013).

Investigation into the mechanism whereby the *Drosophila* ortholog for the mammalian neuropeptide Y (NPY), short neuropeptide F (sNPF) modulates metabolism and lifespan revealed an up-regulation of *dilp1* and *dilp2* mRNA as the result of sNPF overexpression accompanied by increased IIS in the periphery (Lee et al., 2008). As mammalian NPY positively regulates food intake, those results provide additional evidence linking nutrient status and *dilp* levels. A recent study by Yu et al. demonstrated that dCbl (Casitas B-lineage lymphoma), a member of *Drosophila* E3 ubiquitin ligases and adaptor proteins, negatively regulates the *expression of* brain *dilps*. Neuronal and IPC-specific knockdown of *dcbl* results in up-regulation of *dilps 2, 3, 5* whereas the Epidermal growth factor receptor (EGFR) signaling pathway mediates this regulatory effect of dCbl only on *dilps 2* and *3.* Thus, a likelihood of other mediators for *dilp5* is speculated (Yu et al., 2012).

Interestingly, unlike *dilp3* and *dilp5* whose expression levels are suppressed upon starvation, *dilp6* transcript levels are induced under nutrient deprivation and dFOXO is shown to modulate this response in larvae (Slaidina et al., 2009). During late larval and pupal stages when animals cease to feed, *dilp6* expression is strongly induced (Okamoto et al., 2009; Slaidina et al., 2009). As this high level of *dilp6* expression during the larval-pupal transition coincides with a surge of the hormone ecdysone, Slaidina et al. indeed demonstrated that *dilp6* transcription is induced by high levels of ecdysone in the fat body and is required for growth prior to metamorphosis (Slaidina et al., 2009). Fat body specific expression of dFOXO down-regulates *dilp2* which is mediated by DILP6 (Bai et al., 2012). Although basal levels of ecdysone regulates growth through dFOXO during larval development (Colombani et al., 2005), late stage larval expression of *dilp6* could be induced by ecdysone in *dFOXO RNAi* larvae, indicating that regulation of *dilp6* expression by ecdysone acts independently of dFOXO (Slaidina et al., 2009). In addition, *dilp6* also regulates the expression of *dilp5*, when over-expressed in the adult fat body (Bai et al., 2012).

MicroRNAs (miRNAs) play a prominent role in regulating insulin secretion in β-pancreatic cells (Poy et al., 2004). One such miRNA, miR-14 expressed in *Drosophila* IPCs systemically regulates fat levels. Using a reverse genetic approach, Varghese et al. detected reduced *dilp3* and *dilp5* mRNA levels in miR-14 mutant flies, which accompanied increased triglyceride levels (Varghese et al., 2010). Interestingly, the hyperlipidemic defect seen in miR-14 mutants was rescued by overexpressing *dilp3* implying that miR-14 regulates lipid metabolism through modulation of *dilp3* and also outlines a role for *dilp3* in this regard (Varghese et al., 2010). Another miRNA found in the fat body, miR-278 acts to improve insulin sensitivity. *miRNA-278* knockout flies had elevated transcript levels of *dilps2, 3, 5* and also had higher circulating levels of trehalose indicating a condition akin to insulin resistance (Teleman et al., 2006). The involvement of miRNAs in regulating insulin response in the fat body as well as *dilp* expression in IPCs provide exciting evidence for the complexity of selective *dilp* regulation that warrants further investigation.

There is a marked distinction between regulation of *dilp* expression and DILP secretion, as regulatory mechanisms exist in controlling the release of the DILPs. For example, while initial characterization of *dilp* expression pattern affected by diet conditions showed down regulation of *dilp3* and *dilp5* expression but not *dilp2* under starvation (Ikeya et al., 2002), recent availabilities of DILP antibodies made it possible to detect the accumulation of DILPs in IPCs as an indirect measure of DILP secretion. Interestingly, increased accumulation of DILP2 and DILP5 was measured in IPCs as the result of poor nutrient diet or starvation despite unchanged *dilp2* mRNA levels (Geminard et al., 2009). Thus, understanding regulatory mechanisms that modulate DILP secretion should provide more physiological relevance of DILP action. Indeed, cell non-autonomous control of DILP secretion from the IPCs has been identified. NS3, a *Drosophila* nucleostemin family GTPase in the serotonergic neurons is shown to regulate DILP2 levels in the neighboring IPCs, establishing a possible communication between the two neuronal systems (Kaplan et al., 2008) and that an increased accumulation of DILP2 in IPCs and decreased peripheral insulin signaling measured in *ns3* mutants strongly suggested impairment in DILP2 secretion (Kaplan et al., 2008). Soon after this report, Geminard et al. demonstrated a distinct mode of long distance control network of DILP secretion between the fat body and IPCs (Geminard et al., 2009). In this study, it was elegantly demonstrated through *ex vivo* tissue co-culture experiments that the abdominal fat body, functionally homologous to mammalian liver and white adipose tissue and acting as a nutrient sensor, relays this information to brain IPCs by a hormonal signal that involves target of rapamycin (TOR) signaling (Geminard et al., 2009). This hormonal signal emanating from the larval fat body regulates the secretion of DILP2 and DILP5 from the brain IPCs according to the nutrition state (Geminard et al., 2009). Consistent with the notion that fat body relays this secretory signal to the IPCs through a hormone it releases, Unpaired 2 (Upd2), a cytokine produced by the fat body was recently shown to fulfill this role (Rajan and Perrimon, 2012). Upd2 senses the fed state and regulates DILP2 and DILP5 secretion from brain IPCs where under a fed state, there is less DILP accumulation in the IPCs indicating increased DILP secretion. As expected, flies with *upd2* knockdown exhibited increased DILP accumulation under a fed state, illustrating an inability of IPCs to respond to insulin demands (Rajan and Perrimon, 2012). Thus, Upd2 appears to be an important factor regulating DILP secretion from the IPCs.

A recent study by Bai et al. identified that DILP6, produced by the fat body could act as another regulatory factor directly or modulate other factors to affect levels of circulating DILP2 (Bai et al., 2012). Both *dilp2* transcripts in IPCs and circulating DILP2 peptides are reduced in flies overexpressing *dilp6* in the abdominal fat body. This effect appears to be specific to DILP2 as little change in circulating DILP5 levels was observed. Therefore, DILP6 cell non-autonomously decreases IIS by presumably serving as an adipokine or potentially regulating a downstream adipokine that represses *dilp2* expression in the brain IPCs and its secretion (Bai et al., 2012). This study has provided another important piece of evidence of how the abdominal fat body may influence systemic IIS by controlling DILP2 secretion from the IPCs.

**Figure 1:**
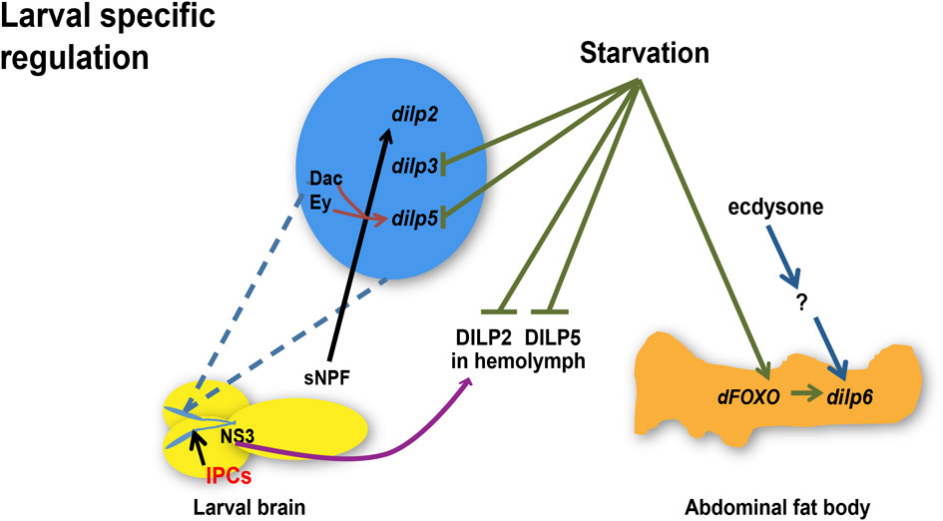
**Regulation of *dilp* expression and circulating DILP levels in response to nutrient status, transcriptional factors, hormone, and a neuropeptide during larval development.** Starvation represses *dilp3* and *dilp5* expression and induces *dilp6* expression through dFOXO in the abdominal fat body whereas circulating DILP2 and DILP5 levels in the hemolymph are diminished. Ecdysone regulates *dilp6* during larval-pupal transition through unknown effectors. sNPF peptide secreted from sNPFnergic neurons located adjacent to IPCs increases *dilp2* expression. NS3 in serotonergic neurons positively regulates DILP2 secretion. Transcriptional factors Dachshund (Dac) and Eyeless (Ey) synergistically promote the expression of *dilp5*. *dilps,* genes encoding *Drosophila* insulin-like peptides; DILPs, *Drosophila* insulin-like peptides; IPCs, Insulin-like peptide producing cells; NS3, a nucleostemin family GTPase; sNPF, short neuropeptide F. Arrows indicate positive regulation whereas blunt-ended lines indicate negative regulation.

**Figure 2:**
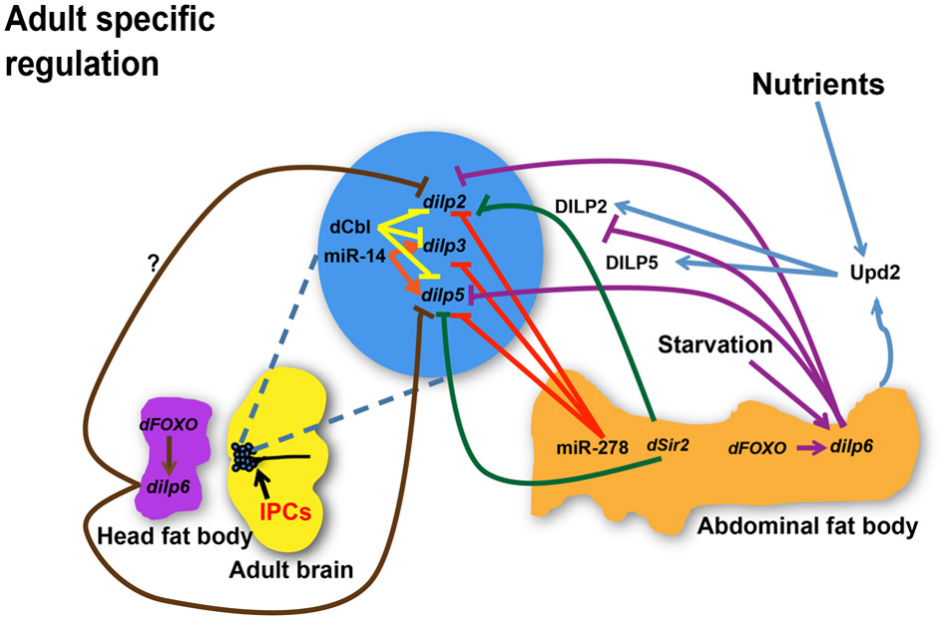
**Regulation of *dilp* expression and circulating DILP levels in response to nutrient status, micro RNAs (miRNAs), and transcriptional factors during adulthood.** miR-278 down-regulates *dilps* expressed in the IPCs while miR-14 positively regulates *dilp3* and *dilp5* expression. dCbl in the IPCs down-regulates *dilps 2, 3,* and *5*. *dSir2* in the fat body represses expression of *dilp2* and *dilp5*. dFOXO acts through *dilp6* in the abdominal fat body to repress *dilp2* while it is not known if dFOXO in the head fat body regulates *dilp2* through a similar mechanism. *dilp6* in abdominal or head fat body tissues negatively regulates *dilp2* and *dilp5.* While *dilp6* only impairs hemolymph DILP2 levels, Upd2 senses the fed state and induces secretion of DILP2 and DILP5. *dilps,* genes encoding *Drosophila* insulin-like peptides; DILPs, *Drosophila* insulin-like peptides; IPCs, Insulin-like peptide producing cells; dCbl, a member of E3 ubiquitin ligases and adaptor proteins; *dSir2*, a histone deacetylase. Arrows indicate positive regulation whereas blunt-ended lines indicate negative regulation.

Taken together, current studies suggest that temporal and spatial transcriptional regulation of major *dilps* is controlled by NS3, sNPF, ecdysone, and dFOXO in larval stages (Figure 1) whereas miRNAs, dFOXO, dSir2, Upd2, and dCbl are regulatory molecules involved in *dilp* transcriptional control in adult (Figure 2). Additional influences of nutrient status are likely to further contribute to differential regulation of *dilp2, 3*, *5,* and *6*, which predicts diverse functionality of each DILP in mediating IIS under diverse physiological environments.

## Functions of DILPs

Analogous to the opposing actions of insulin-secreting β-pancreatic cells and glucagon-secreting pancreatic islet α-cells in maintaining glucose homeostasis in mammals, IPCs and adipokinetic hormone (AKH)-producing corpora cardiaca (CC) cells are neurosecretory cells that function to regulate metabolic processes in the fly (Rulifson et al., 2002; Kim and Rulifson, 2004). Studies of neurosecretory network in larval brain have detected DILP2, but not *dilp2* mRNA in CC cells suggesting an undefined role of DILP2 in CC cells, away from the site of production, IPCs (Rulifson et al., 2002). Whether there is a similar movement of DILP3 or DILP5 from IPCs to CC cells remains to be determined. An interesting piece of evidence stems from the fact that adult IPCs respond to glucose or trehalose feeding as well as a K_ATP_ channel blocker with an increase in Ca^2+^ influx and membrane depolarization, which provides indirect evidence for the presence of ATP sensitive K^+^ channels in those neurosecretory cells similar to those found in β-pancreatic cells in mammals (Fridell et al., 2009).

The first set of compelling evidence demonstrating the functional extent of DILPs in controlling growth, development, and glucose homeostasis was generated by the destruction of IPCs. Ablation of IPCs during the early larval stage results in severe developmental delay with a reduction of both cell numbers and body size accompanied by an increased level of circulating sugars suggesting a diabetic-related phenotype (Rulifson et al., 2002). Importantly, a partial rescue of growth and circulating sugar phenotypes with *dilp2* overexpression strongly supported the notion that loss of DILP2 was responsible for the phenotypes (Rulifson et al., 2002). Ablation of IPCs in late larval stages results in a minor developmental delay and slightly decreased body size (Ikeya et al., 2002), reduced fecundity, higher energy stores of lipids and carbohydrates and an extended lifespan (Broughton et al., 2005). It was later demonstrated by Buch et al. that reduced fecundity was dissociated from the longevity effect, as flies with post-larval IPC ablation are long-lived on a protein-rich diet with normal fecundity (Buch et al., 2008). Similar to the larval effects on glucose homeostasis, adult-specific partial ablation of IPCs renders flies hyperglycemic and glucose intolerant but insulin sensitive as measured by peripheral glucose disposal upon insulin injection and serine phosphorylation of a key insulin-signaling molecule, Akt (Haselton et al., 2010). In addition, a significant increase in stored glycogen and triglyceride levels as well as an elevated level of circulating lipids was measured in adult IPC knockdown flies with an extended lifespan thus demonstrating that it is possible to modulate DILP action in adult flies to achieve lifespan extension without insulin resistance. With the development of an oral glucose tolerance test in the adult fly, this report documented that adult IPCs indeed are responsible for executing an acute glucose clearance response (Haselton et al., 2010). While this study clearly demonstrates profound metabolic and longevity phenotypes as the result of impaired DILP-producing IPCs in an adult-specific manner, it remains to be determined the specific involvement in metabolism and aging of each DILP produced in IPCs.

## Disruption of DILPs in lifespan regulation

The role of IIS pathway in aging was first discovered when mutations of *daf-2*, a gene encoding the insulin receptor homolog in *C. elegans* nearly doubled the lifespan of the organism (Kenyon et al., 1993). Mutations disrupting IIS molecules such as DInR or the *Drosophila* homolog of the insulin receptor substrate CHICO similarly render cell non-autonomous effects in lifespan extension as the result of reduced IIS (Clancy et al., 2001; Tatar et al., 2001). Genetic manipulation of expression of additional IIS components such as overexpression of dFOXO in the abdominal or pericerebral fat body or dPTEN in the pericerebral fat body mimicking reduced IIS is sufficient to extend lifespan (Giannakou et al., 2004; Hwangbo et al., 2004). Likewise, partial ablation of IPCs, the production site of DILPs 2, 3, and 5 reduced IIS and recapitulated the longevity phenotype when starting in late larval stage (Broughton et al., 2005) or in an adult-specific manner (Haselton et al., 2010). However, the role of individual DILPs in controlling the aging process has proven difficult to ascertain due to functional redundancy and compensation among DILPs.

A significant amount of interest has been bestowed upon DILP2 as its transcript is most abundantly expressed among all *dilps* and DILP2 possesses the highest homology to the mammalian insulin with a 35% identity in protein sequence (Brogiolo et al., 2001). Down-regulation of *dilp2* is associated with lifespan extension under several conditions. First, activation of dFOXO in the pericerebral fat body extends lifespan with an accompanied reduction in *dilp2*, but not in *dilp3* or *dilp5* mRNA levels (Hwangbo et al., 2004). Second, upon JNK (Jun-N-terminal kinase) activation in the IPCs, dFOXO-dependent repression of *dilp2* is associated with the extension of lifespan observed in those flies (Wang et al., 2005). Third, expression of a dominant negative form of p53 in adult neurons extended lifespan and reduced *dilp2* transcript levels, again indicating that the reduction of *dilp2* expression was closely associated with extended longevity under those genetic conditions (Bauer et al., 2007). Although those results indicate a close association between decreased *dilp2* expression and increased lifespan, direct modulation of *dilp2* levels was needed to assess the causal relationship between *dilp2* expression and lifespan control. To this end, surprisingly, while causing a severe reduction of *dilp2* transcripts, targeted knockdown of *dilp2* in IPCs did not result in any lifespan extension (Broughton et al., 2008). But interestingly, an increase in *dilp3* and *dilp5* expression was observed in those flies raising the possibility that a compensatory mechanism exists to modulate overall *dilp* expression in the IPCs. However, this compensatory increase in *dilp3* and *dilp5* expression could not completely account for the lack of lifespan extension in *dilp2* knockdown flies as a similar increase of *dilp3* and *dilp5* transcripts was observed in long-lived *dilp2* null mutant flies and increased *dilp5* expression levels in long-lived *dilp2–3* mutants (Gronke et al., 2010). Thus, it remains possible that *dilp2* knockdown elicits additional genetic alterations neutralizing the effect on lifespan associated with reduced *dilp2* transcripts. The extended lifespan measured in *dilp2* null mutants, however, confirms a major role of DILP2 in longevity control. The absence of any change in lifespan in flies with a *dilp3* deletion is intriguing as both *dilp2* and *dilp5* transcript levels are lowered in those flies (Gronke et al., 2010). A lack of consistent correlation between *dilp* transcript levels and lifespan effects in *dilp2, dilp2–3,* and *dilp3* null mutants requires further clarification with measurements of DILP peptide levels as possible compensatory mechanisms to modulate IIS and lifespan regulation. An involvement of DILP3 in longevity control is worth further investigation, however as *dilp3* transcript levels appeared to be specifically reduced in long-lived flies with increased mitochondrial uncoupling in adult IPCs (Fridell et al., 2009). While a *dilp5* null mutant appeared to have no effect on lifespan under standard diet (Gronke et al., 2010), *dilp5* levels are moderated in DR-mediated lifespan extension (Min et al., 2008) (discussed below). A *dilp6* loss-of-function mutation neither had any effect on adult *Drosophila* survival nor on any compensatory increase in the expression of other *dilps* (Gronke et al., 2010). On the other hand, Bai et al. recently showed that overexpressing *dilp6* in the adult abdominal fat body significantly extends lifespan in females in a diet-dependent manner and negatively affects expression of *dilp2* and *dilp5*, whereas a modest effect in survival is observed when *dilp6* is expressed in the pericerebral fat body. This study also shed light on the fact that the longevity effect of dFOXO when overexpressed in the pericerebral fat body requires *dilp6* (Bai et al., 2012). Taken together, creation of individual or combinatorial *dilp* mutants has confirmed lifespan extension as the result of *dilp2* deficiency suggesting a major role of DILP2 in modulating IIS. On the other hand, targeted expression of *dilp6* in the adult fat body results in profound longevity and metabolic consequences that underlies its role in lifespan regulation.

To aid a better understanding of the significance of DILP2 and DILP6, physiological alterations that accompanied lifespan extension in respective *dilp* mutants have paved the way. IPC ablated flies exhibit high levels of trehalose, lipid and glycogen stores, accompanied by increased stress resistance (Broughton et al., 2005). With respect to DILP2, the phenotypic changes as a result of its down-regulation were associated with higher trehalose storage levels and slight resistance to starvation (Broughton et al., 2008). Increased trehalose levels were also seen in a *dilp2* loss-of-function mutant with no change in lipid or glycogen levels (Gronke et al., 2010). Nevertheless, those findings imply a role for DILP2 in trehalose metabolism, which may explain a moderate starvation resistance in those flies. *dilp 1–4* loss of function mutants were starvation resistant recapitulating the role for *dilp2* in starvation resistance (Gronke et al., 2010). Surprisingly, neither *dilp2* null mutants nor *dilp 2–3, 5* deletion mutants, created by homologous recombination, were resistant to starvation (Gronke et al., 2010). The evidence that IPCs, independent of insulin signaling, mediate response to starvation (Mattaliano et al., 2007) could possibly account for the starvation resistance in IPC ablated flies (Broughton et al., 2005) and the lack of starvation response in *dilp2* null mutants. A putative role for DILP2 in response to oxidative stress was discovered in the context of JNK signaling upon oxidative stress where *dilp2* expression is repressed in IPCs (Wang et al., 2005). However, neither the *dilp2 RNAi* hypomorphs (Broughton et al., 2008) nor the *dilp2* loss-of-function mutants (Gronke et al., 2010) displayed any resistance to oxidative stress, excluding a direct role for DILP2 in response to oxidative stress. These studies thus, point to a role for DILP2 in trehalose metabolism, which could contribute to lifespan extension as the result of increased energy storage. While adult flies harboring *dilp6* over expression in the abdominal fat body exhibit metabolic phenotypes reminiscent of those seen as a consequence of reduced IIS (Bai et al., 2012), *dilp6* loss-of-function mutants only had high stored lipid levels revealing its specific role in lipid storage (Gronke et al., 2010). This is substantiated by the fact that DILP6 plays an important role in reallocating energy stores during the non-feeding pupal stage in preparation for metamorphosis (Slaidina et al., 2009).

Unlike DILP2 and DILP6, an involvement of DILP3 and DILP5 in any physiological feature that plays a part in lifespan regulation has not been identified. *dilp3* or *dilp5* single mutants were neither resistant to starvation or oxidative stress nor was there any change in their trehalose, glycogen, or lipid levels (Gronke et al., 2010), although *dilp3* overexpressors play a role in regulating triglyceride levels (Varghese et al., 2010).

## Modulation of DILPs under dietary restriction

Through dilution of nutrient content, DR is a robust intervention that has been shown to extend lifespan in *Drosophila*. While the exact molecular mechanisms behind DR-mediated lifespan extension are yet to be completely elucidated, several molecular pathways have emerged as important players involved in DR responses (Narasimhan et al., 2009). Within the scope of this review, we will discuss current understanding of the involvement of IIS cascades or DILPs in DR. Interestingly, in *C. elegans*, depending upon the methods of DR, lifespan extension associated with DR is largely independent of IIS (Kaeberlein et al., 2006; Lee et al., 2006; Bishop and Guarente, 2007; Smith et al., 2008). Similarly, IIS-dependent and IIS-independent mechanisms exist in DR-associated lifespan extension in *Drosophila* (Clancy et al., 2002; Min et al., 2008). While Clancy et al. demonstrated that long-lived *chico* mutants did not respond to optimal DR for additional lifespan extension indicating an overlap between IIS and DR (Clancy et al., 2002), Min et al. showed that *dFOXO* mutants remained sensitive to DR thus suggesting that DR acts independently of IIS (Min et al., 2008). A potential explanation for this discrepancy may be the different DR regimens used in those studies. An overall dilution in diet was used in (Clancy et al., 2002) whereas reducing yeast concentration to achieve DR was employed in Min et al. (2008). Thus, future studies should aim at standardizing DR conditions in *Drosophila* in order to reconcile discrepant findings as well as pinpoint a role of IIS in DR (Tatar, 2011).

There is emerging evidence on the role of IPCs in lifespan extension through DR, as those neurosecretory cells appear to respond to nutrient changes (Broughton et al., 2010). With regard to DILPs, DR conditions in *Drosophila* are shown to extend lifespan with changes in *dilp5* mRNA levels but not *dilp2* or *dilp3* levels (Min et al., 2008). Both *dilp5* mRNA and DILP5 protein levels are down-regulated in wild type flies under a yeast DR diet where only yeast is diluted while keeping carbohydrate levels constant (Broughton et al., 2010). Hence, DILP5 may serve as a central cue in understanding the molecular mechanisms behind DR.

Flies with *dilp5* knocked down by a UAS-*dilp3RNAi* construct that repressed expression of *dilps 2, 3,* and *5* and blocked the nutrient-dependent expression of *dilp5*, exhibited a normal response to DR under a yeast DR regime implying that DR-mediated lifespan extension works independently of DILP5 (Min et al., 2008). Whereas overexpression of *dilp6* in the fat body lowers *dilp2* and *dilp5* expression as well as the respective hemolymph peptide levels, the lifespan of *dilp6* overexpressors was similar to the controls under a yeast restricted diet corroborating with the evidence that DR works independently of *dilp5* and DILP5 (Bai et al., 2012).

However, *dilp5* null mutant flies that displayed a normal DR response also exhibited a compensatory up-regulation of *dilp2* mRNA when raised on food with high yeast concentration while *dilp3* mRNA levels were up-regulated in these flies raised on food with relatively low yeast concentration (Gronke et al., 2010). Thus, it raises the possibility that compensatory transcriptional regulation could negate any change in lifespan in *dilp5* loss-of-function flies raised on yeast DR diet. Supporting evidence for the involvement of DILPs in DR, if not a direct role, was presented when *dilp 2–3, 5* deletion mutant flies (Gronke et al., 2010) or IPC ablated flies (Broughton et al., 2010) on yeast DR diet exhibit an atypical DR response. These results hint at a potential mechanism in DR involving DILPs as with dFOXO which is not required for DR *per se* but whose activity has shown to modulate DR response when over-expressed (Giannakou et al., 2008), a scenario for an indirect or a secondary role of DILP5 in DR remains possible. Alternatively, while *dilp5* is modulated under DR, this change in expression could simply be a response associated with dietary alterations but does not trigger the longevity effect of DR. Further clarification is required to definitively assign a physiological role of DILP5, if any, in DR response.

## Conclusion and outlook

**Figure 3:**
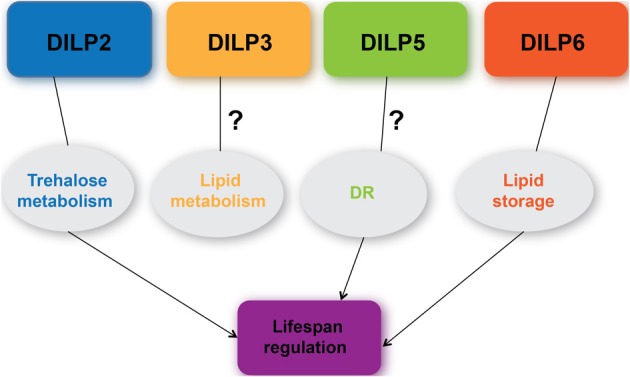
**A current understanding of the roles of DILPs 2, 3, 5, 6 in metabolism, DR response, and lifespan regulation**.

DILPs are involved in a myriad of physiological processes ranging from growth (Brogiolo et al., 2001; Rulifson et al., 2002; Slaidina et al., 2009; Colombani et al., 2012; Garelli et al., 2012), metabolism (Broughton et al., 2008; Gronke et al., 2010; Bai et al., 2012), to lifespan (Broughton et al., 2005; Gronke et al., 2010; Bai et al., 2012) (Figure 3). This review that has focused on DILPs 2, 3, 5, and 6 has highlighted some of the regulatory mechanisms governing their expression and secretion, and their functions pertaining to lifespan regulation as well as the controversy surrounding the role of DILPs in DR. Compensatory transcriptional regulatory mechanisms and functional redundancy that exist among DILPs make it difficult to dissect out their individual roles. A similar functional redundancy is observed where the *Drosophila* homolog of IGF-binding protein, Imp-L2 is shown to bind to DILP2 (Honegger et al., 2008), although DILP6 is most similar in structure to vertebrate IGF (Okamoto et al., 2009). Nonetheless, genetic approaches that have targeted tissue specific expression or disruption of individual *dilp*s have confirmed that loss of *dilp2* and over-expression of *dilp6* is sufficient to extend lifespan (Gronke et al., 2010; Bai et al., 2012). Although a direct role for DILP5 in DR-mediated lifespan extension remains controversial, its involvement cannot yet be entirely excluded. In addition, information garnered from *Drosophila* as a model to study cross talk between the nutrient sensing fat body and the neurosecretory IPCs has shed significant insight into a systemic control of DILP activities as the result of communication between those two tissues tightly associated with metabolism. The studies highlighted in this review have underscored the importance of measuring DILP levels in order to substantiate and validate their functional significance. Specifically, measuring circulating DILPs in the hemolymph should provide most relevant assessment on secreted DILP levels and their systemic effects (Bai et al., 2012).

Ablation of IPCs or deletion of *dilps 1–5* mimics phenotypes seen in type 1 diabetes (Rulifson et al., 2002; Zhang et al., 2009) while insulin resistance seen in flies fed a high sugar diet are associated with modulated *dilp* levels in modeling type 2 diabetes (Musselman et al., 2011; Morris et al., 2012). Thus, through genetic modulation of *dilps* in *Drosophila*, metabolic disorders such as diabetes, hyperinsulinemia, or those affecting glucose homeostasis can be modeled in this genetic organism. These approaches will likely further characterize the molecular mechanisms behind these disorders, discover drug targets, and screen potential therapeutic modes to treat these disorders. Apart from disease models, emerging research has revealed an involvement of DILPs in stem cell biology including stem cell proliferation (LaFever and Drummond-Barbosa, 2005; Sousa-Nunes et al., 2011), reactivation of neural stem cells from their quiescent stage (Chell and Brand, 2010) and germ-line stem cell maintenance (Hsu and Drummond-Barbosa, 2009). Recent findings on the differential expression of *dilp8* in tumor eye discs that responds to signals from peripheral tissues to mediate their growth and development has further strengthened *Drosophila* as a model for investigating mechanisms underlying inter-organ communication and demonstrated a role for DILP8 in cancer biology (Garelli et al., 2012). Overall, DILPs, as outlined in the review, contribute to growth and development, metabolic homeostasis, and longevity regulation in *Drosophila* through diverse mechanisms that are being unraveled.

### Conflict of interest statement

The authors declare that the research was conducted in the absence of any commercial or financial relationships that could be construed as a potential conflict of interest.
